# Understanding the experiences of young, urban, Indigenous mothers-to-be in British Columbia, Canada

**DOI:** 10.1186/s12884-024-07070-1

**Published:** 2025-01-20

**Authors:** Nicole L. A. Catherine, Jennifer Leason, Namaste Marsden, Brittany Barker, Ange Cullen, Ashley Simpson, Brandi Anne Berry, Erik Mohns, Donna Yung, Yufei Zheng, Harriet MacMillan, Charlotte Waddell

**Affiliations:** 1 Faculty of Health Sciences, Simon Fraser University, Vancouver, BC Canada; 2 Department of Anthropology and Archaeology, University of Calgary, Calgary, AB Canada; 3 Gitanyow Nation; Faculty of Health Sciences, Simon Fraser University, Burnaby, BC Canada; 4 First Nations Health Authority, Vancouver, BC Canada; 5 Offord Centre for Child Studies, Departments of Psychiatry and Behavioural Neurosciences, Faculty of Health Sciences, McMaster University, Hamilton, ON Canada; 6 Children’s Health Policy Centre, Faculty of Health Sciences, Simon Fraser University, Room 2435, 515 West. Hastings Street, Vancouver, B.C V6B 5K3 Canada

**Keywords:** Indigenous, Girls, Women, Adolescents, Mothers, Socioeconomic disadvantage, Pregnancy

## Abstract

**Background:**

Indigenous Peoples comprise the youngest and fastest growing demographic in Canada, with many living in urban-suburban areas. Given higher fertility rates, younger overall ages and higher adolescent pregnancy rates, perinatal research is needed—to inform policymaking and programming throughout pregnancy and childhood. Yet such data remain scarce in British Columbia (BC), Canada. This study therefore aimed to describe the experiences of young, urban, Indigenous mothers-to-be who enrolled in a larger BC early prevention trial designed to reach families experiencing socioeconomic disadvantage.

**Methods:**

This descriptive study utilized baseline data from a trial that enrolled first-time mothers-to-be who met indicators of socioeconomic disadvantage and who were residing in select urban-suburban areas. These indicators included being young (19 years or younger) or having limited income, low access to education, and being single (aged 20−24 years). We described and compared survey data on girls (*n* = 109; aged 14−19 years) and young women (*n* = 91; aged 20−24 years) using Chi-square or Student’s *t*-tests.

**Results:**

Of the 739 trial participants, 200 or 27% identified as Indigenous and met trial eligibility criteria: limited income (92.9%), limited access to education (67.0%), and/or being single (90.9%). Beyond this, participants reported associated adversities including: unstable housing (63.3%), psychological distress (29.3%), severe anxiety or depression (48.5%), experiences of childhood maltreatment (59.4%) and intimate partner violence (39.5%). Compared to girls, young women reported higher income and educational attainment (*p* < 0.001), more unstable housing (*p* = 0.02) and more childhood maltreatment (*p* = 0.014). Many had recently received primary healthcare (75%), but few had received income assistance (34%). Most (80.5%) reported experiencing four or more adversities.

**Conclusions:**

We present data illustrating that a high proportion of pregnant Indigenous girls and young women engaged with public health and consented to long-term research participation—despite experiencing cumulative adversities. The trial socioeconomic screening criteria were successful in reaching this population. Girls and young women reported relatively similar experiences—beyond expected developmental differences in income and education—suggesting that adolescent maternal age may not necessarily infer risk. Our findings underscore the need for Indigenous community-led services that address avoidable adversities starting in early pregnancy.

## Background

In Canada, more than 1.8 million people identified as Indigenous in 2021 (defined as First Nations, Métis or Inuit), comprising 5.0 per cent of the population [1]. There are more than one million First Nations, 624,000 Métis and 70,000 Inuit [1]. Indigenous groups have distinct histories, cultures, worldviews and traditions, with more than 70 languages [2]. Most Indigenous Peoples (82.1%) live “off-reserve” (i.e., not in a place that is part of a federal government census subdivision designation labelled as “on reserve”) [1]. The Indigenous population is also the youngest and the fastest growing demographic in Canada—expected to climb to nearly 3.2 million people (6.8% overall Canadian population) over the next three decades [3]. Compared to non-Indigenous children in Canada, Indigenous children under age five years form a higher proportion of the population (7.6% versus 5.0%), with mean overall ages being younger (33.6 years versus 41.4 years) [1]. Population increases are attributed to increased self-identification as well as higher fertility rates [3]. As well, more Indigenous adolescents (10.5%) experience pregnancy and become young mothers compared to non-Indigenous adolescents (2.9%) [4]. Given higher fertility rates, the younger overall demographic and higher adolescent pregnancy rates, it is important to understand the experiences of young Indigenous mothers-to-be, to inform policy changes that can improve wellness throughout pregnancy, early childhood and beyond. Yet few such data are available in British Columbia (BC), Canada.

In Canada and elsewhere, the healthy development of Indigenous children—including teaching traditional values, principles and Ways of Knowing and Being—is crucial not only for young people but also for the health and well-being of Indigenous communities. [5, 6] Indigenous Peoples have diverse and longstanding strong and nurturing cultural practices that support healthy families and child development. According to many Indigenous belief systems, the period from pregnancy through early childhood is also considered sacred, with children being considered gifts from the Creator—the future of Indigenous Peoples. [7, 8] Traditionally, expectant mothers therefore received culturally-grounded support and wisdom from family, midwives and community members. [8, 9] However, these Traditional Knowledges and practices have been systematically eroded for generations through harmful government policies and practices, resulting in disproportionate rates of adverse health outcomes for First Nations, Métis and Inuit mothers and children [10–12]. 

Globally, Indigenous Peoples experience poorer health outcomes in comparison with their non-Indigenous counterparts due to ongoing legacies of colonialism and systemic racism. [13, 14] Despite being a high-income country, Canada is no exception, with Indigenous Peoples continuing to experience higher rates of chronic disease, lower rates of self-reported mental health, and lower life expectancy [15–17]. In Canada, the federal government is responsible for health services “on reserve”, while provincial and territorial governments cover those living “off reserve.” The exception is BC where the First Nations Health Authority (FNHA) has assumed responsibility and leadership for public health and healthcare across the province [18]. Indigenous mothers-to-be in Canada also experience greater disparities in perinatal and infant health outcomes in comparison to non-Indigenous mothers and infants [11, 19–21]. Previous research has found these adverse events and outcomes are largely associated with inadequate access to healthcare, particularly in remote communities [11, 22]. Health personnel are lacking for many communities, as are culturally-appropriate supports, information and resources [23, 24]—demonstrating how mothers-to-be experience varying levels of power, choice and control over their bodies and their pregnancies. Mothers and their children also experience provider discrimination, structural racism and harm in healthcare [25, 26] and avoid accessing healthcare when family policing or child placement in out-of-home care is being threatened [27–30]. This fear is attributed to Canada’s recent history of genocidal health policies and practices such as the forced and coerced sterilization of girls and women [31, 32] or the practice of ‘birth alerts’ that to this day, despite being formally disallowed, may lead to forced maternal-child separation [33]. 

In Canada, beyond unequal access to health services, poorer Indigenous health outcomes are also the result of social determinants [6, 34]. The origins of these determinants include displacement from ancestral lands and creation of land reserve systems, thereby fracturing traditional economies and governance structures, and legislation that precluded basic human rights such as voting, owning land and practicing cultural ceremonies. [16, 17] Resultant systemic socioeconomic inequities affecting health still include: limited educational opportunities; chronic high unemployment; low incomes; lack of potable water; food insecurity; and overcrowded and unsafe housing. [16, 17, 21, 35] Beyond these socioeconomic determinants, health inequities are also deeply rooted in racism [36, 37]—and the intersection of these forces particularly affects Indigenous women and mothers [25, 38]. Most damaging for children and families, colonial practices included the forced removal of more than 150,000 children from their families and placement into residential schools, occurring across Canada for over a century and interrupting Traditional Practices over many generations. [16, 17] Assimilative and damaging child welfare policies also continue, resulting in disproportionate numbers of children (53.8% of children under 14 in foster care) still being removed from their families and communities [27, 39] with lifelong and intergenerational repercussions [40]. 

Despite these adversities, Indigenous leaders and communities have long persevered, demonstrating the route to resilience, resurgence and reconciliation [6, 9, 41]. For example, in response to persistent and efficacious advocacy by Indigenous leaders [27], the Canadian Human Rights Tribunal urged the government to fully satisfy a $47.8 billion Canadian dollar (CAD) settlement agreement to compensate families for longstanding inequities in children’s services [42]. Another important route to resilience, resurgence, and reconciliation are efforts to ensure the inclusion of data, provided by Indigenous mothers-to-be, in health and wellness research, while avoiding making comparisons with non-Indigenous study participants [20, 43]. Here, we therefore describe the experiences of young, urban-Indigenous mothers-to-be who enrolled in a larger BC early prevention trial. Upon examination of the trial’s baseline interview data, we retrospectively found that 200 of 739 or 27% of maternal participants identified as Indigenous [44]. Wanting to learn from Indigenous participants and to share their experiences through their survey data, our goal was to describe and compare baseline data on girls (age 14−19 years) and young women (age 20−24 years) who were pregnant and preparing to parent for the first time.

## Methods

### Study design and setting

This descriptive study used baseline data collected from a larger trial, the British Columbia Healthy Connections Project (BCHCP). The BCHCP was an evaluation of an early prevention program designed to support mothers-to-be who were experiencing socioeconomic disadvantage in early pregnancy. This paper reports on descriptive baseline data only (i.e., collected prior to random allocation to treatment groups) and does not report on trial evaluation outcomes. The trial enrolled participants who were residing in 26 local health areas (LHAs) across four of five regional health authorities (HA) including Fraser, Interior, Island and Vancouver Coastal Health. Recruitment and baseline data collection spanned three years, from 2013 to 2016. (One urban LHA in Northern Health was involved in an adjunctive process evaluation [45] but did not participate in the trial.)

The trial, including referral and recruitment processes, was embedded within BC’s public health system—involving active, sustained and reciprocal research-policy-practice collaborations. The trial was governed by a Steering Committee involving senior members of the BC Ministries of Health, Children and Family Development, and Mental Health and Addictions and BC-based scientific team members (NC, CW and HM). The trial received expert policy and practice guidance and support from a Provincial Advisory Committee including the FNHA (AS, NM) and Mètis Nation of British Columbia or MNBC. However, the trial was not developed or designed for Indigenous families. Detailed trial information is described in the published study protocol [46]. Trial results with the full cohort are published elsewhere [47, 48].

### Study participants, recruitment and procedures

For the BCHCP, participants were young (< 25 years), first-time mothers experiencing socioeconomic disadvantage. (Table 1 shows the eligibility criteria.) There were no specialized recruitment pathways for Indigenous participants. When we commenced this study, BC still lacked a harmonized or integrated approach to obtaining research ethics approvals from the 200 First Nations across the province. The FNHA was also still in formation when we started this study. Therefore, we required all participants to be living “off reserve” prior to enrollment.

The four participating health authorities were responsible for recruitment through public health prenatal registries. In BC, pregnant individuals, including urban-Indigenous Peoples, can access public health directly via these registries or referrals from primary healthcare providers such as physicians, nurse practitioners, midwives, or youth-serving agencies including schools [49]. Once referred, public health nurses offered existing services. For the trial, public health nurses also applied the eligibility criteria to then screen and refer potentially-eligible participants to the study team located at the Children’s Health Policy Centre, Faculty of Health Sciences, Simon Fraser University (SFU). The study team contacted potential participants by telephone to describe the research, answer questions, screen for eligibility and schedule an in-person baseline interview. Field interviewers then met with participants in their homes to establish rapport, facilitate trust and describe the details and importance of study engagement given that trial participation was up to 2.5 years in duration. These interviewers received intensive training on participant-centred engagement and retention approaches as well as on the study protocol [46]. Following written informed consent, interviewers collected baseline questionnaire data and provided participants with one gift card as honoraria (worth $75 CAD). For the larger trial, the study team followed them until their first child(ren) reached age two years [46]. 

**Table 1:** Eligibility criteria^*^

Inclusion criteria
• Aged 24 years or younger • Preparing to parent for the first time^†^ • Less than 28 weeks gestation • Competent to provide informed consent^#^ • Experiencing socioeconomic disadvantage: ° Aged 19 years or younger, or, ° Aged 20−24 years and having two or more of the following indicators: • Living on a low income (one or more of the following): Receiving Medical Services Plan, disability or other income assistance; finding it difficult to live on total household income, particularly for food or rent; and/or currently homeless^‡^ • Having limited access to education (less than BC high-school diploma) • Preparing to parent while single (not married or in a common law relationship)^±^
**Exclusion criteria**
• Planning to have their child adopted • Planning to leave the BCHCP trial catchment area for three months or longer

* Adapted from Catherine et al., 2020a; 2016. [46, 47] † Girls and young women were eligible if any previous pregnancy ended in termination, miscarriage or stillbirth, or if previous parenting involved step-parenting. # Competence included ability to converse in English. ‡ Homeless was defined as living on the streets, living in a place not meant for permanent dwelling (e.g., car or tent) or living in a shelter or staying somewhere temporarily with no permanent address (e.g., “couch surfing”). ± Common law was defined as living with the same person for more than one year

### Measures

In collaboration with policy and practice partners, the scientific team identified the baseline indicators of socioeconomic disadvantage (i.e., young age, limited income, limited education, being single) that we hypothesized would be associated with the BCHCP trial main outcomes (i.e., child health and development by age two years). These indicators were selected based on relative ease of screening by nurses and field interviewers, rather than screening regarding more sensitive topics such as mental health problems or maltreatment experiences. These indicators also ensured HAs reached and referred the population that the early prevention program was designed to benefit. Yet some outcomes could also be interpreted as capturing strengths, for example, accessing public services.

All baseline data reported here were collected by maternal self-report during computer-assisted in-person interviews. Interviewers read items aloud and recorded participants’ answers. Potentially sensitive items, such as prenatal substance use and maternal maltreatment experiences (during their childhood and within the past year) were completed by participants using audio recordings, before placing their confidential pen-and-paper responses in a sealed envelope for later data processing. For a summary of baseline measures, see Table 2.

**Table 2:** Summary of measures^*^

Living on limited income
• Earning less than $20,000 CAD annually (before taxes) including money earned in self-employment and excluding money received from family, friends or income assistance [50]
Having limited access to education
• Not having equivalent of a British Columbia (BC) high school diploma
Preparing to parent while single
• Neither married nor common-law (i.e., living together consecutively one or more years)
Sociodemographic characteristics
• Indigenous was defined as one of “First Nations, Métis or Inuit”; education; annual income; age [50, 51]
Homelessness
• Living on the streets, in a place not meant for permanent dwelling (e.g., car or tent) or in a shelter; or experiencing “hidden homelessness” by staying somewhere temporarily with no permanent address (e.g., “couch surfing”) [52]
Unstable housing
• Moving three or more times in the past year or ever experiencing homelessness
Psychological distress
• Moderate-to-severe anxiety or depression—total score > 25 using the 10-item Kessler Psychological Distress Scale. [53] Validated among Indigenous adults living off-reserve within Canada [54]
Mental and physical health conditions
• Self-reported, physician-diagnosed health conditions limiting everyday activities including: severe anxiety or depression; diagnosed mental disorder; developmental conditions; and serious long-term physical health conditions [55, 56]
Mental health-substance use
• Frequency of nicotine, alcohol, cannabis and street drug use [51]
Experiences of violence
• Child maltreatment defined as moderate-to-severe neglect, physical abuse, emotional abuse, or and sexual abuse prior < 16 years—using the 28-item Childhood Trauma Questionnaire [57]
• Intimate partner violence in the past year defined as any physical abuse, emotional abuse or harassment—using a score of 7 or more on the 30-item Composite Abuse Scale [58]
Health services for physical health
• Recent primary healthcare provider visits (e.g., family physicians, nurse practitioners, nurses or midwives) and prenatal classes
Social services
• Recent income assistance through provincial or federal programs such as: BC Income and Disability Assistance; Canada Disability Benefits and Employment Insurance; BC Hardship Assistance; or BC Youth Agreements

* Adapted from Catherine et al., 2016; 2019 [44, 46]. CAD = Canadian dollars

### Cumulative adversities

We were also interested in the burden of cumulative adversities that participants might be experiencing in early pregnancy. We therefore assessed the proportion who reported having one or more of the following: (1) living on less than $20,000 CAD annually; (2) having less than a high school education; (3) preparing to parent while single; (4) experiencing unstable housing (three or more moves in the past year or ever homeless); (5) experiencing moderate-to-severe levels of psychological distress; (6) reporting prenatal substance use; (7) having a history of childhood maltreatment when age 16 years or younger; and (8) experiencing intimate partner violence (IPV) in the past year.

### Statistical analysis

Descriptive data were tabulated for the cohort of 200 Indigenous participants, as well as stratified by the two age groups that aligned with the socioeconomic disadvantage eligibility criteria: girls (14–19 years) and young women (20–24 years). For age group comparisons, we then applied Chi-square tests, or the Fisher’s exact test for cell sizes less than five. For continuous variables, we used the Student’s *t*-test. Statistical significance was set at *p* < 0.05. Participants could choose not to respond to items; therefore, in each table row *n* might differ from the overall *n* due to missing data.

## Results

### Indigenous participants

During trial enrollment (2013–2016), 739 participants accessed public health, consented to trial participation and were enrolled in the trial—of whom 200 self-identified during the baseline interviews as having Indigenous ancestry. Of these 200 participants, 39.5% self-identified as exclusively Indigenous while 60.5% self-identified as Indigenous as well as other ancestries. Most (*n* = 99; 49.5%) were residing in Fraser Health Authority in early pregnancy, with others residing in Interior Health (*n* = 38; 19.0%), Island Health (*n* = 31; 15.5%) and Vancouver Coastal Health (*n* = 32; 16.0%) Authorities. See Table 3.

**Table 3:** Number of participants accessing public health and enrolling in study in early pregnancy^*†^

Local Health Area	*n*	%
**Fraser Health Authority**		
Abbotsford	17	8.5
Burnaby	7	3.5
Chilliwack	8	4.0
Coquitlam	10	5.0
Delta	< 5	1.0
Langley	< 5	1.5
Maple Ridge	5	2.5
Mission	8	4.0
New Westminster	< 5	1.0
South Surrey / White Rock	< 5	1.0
Surrey	35	17.5
Fraser Salish Region Total	99	49.5
**Interior Health Authority**		
Central Okanagan	13	6.5
Kamloops	12	6.0
Vernon	13	6.5
Interior Region Total	38	19.0
**Vancouver Island Health Authority**		
Cowichan and Lake Cowichan	< 5	1.5
Greater Victoria	14	7.0
Nanaimo and Ladysmith	10	5.0
Saanich	< 5	0.5
Sooke	< 5	1.5
Vancouver Island Region Total	31	15.5
**Vancouver Coastal Health Authority**		
Richmond	5	2.5
Vancouver – Downtown Eastside	16	8.0
Vancouver – North East	7	3.5
Vancouver – South	< 5	1.0
Vancouver – Westside and Vancouver / Midtown	< 5	1.0
Vancouver Coastal Region Total	32	16.0
**Total**	**200**	**100.0**

* Adapted from Catherine et al., 2020a [47]. † For consistency with the trials’ previous reporting, some smaller local health areas have been combined

### Eligibility criteria and sociodemographic characteristics

Regarding age criteria, 54.5% (*n* = 109) were girls aged 14–19 years and 45.5% (*n* = 91) were young women aged 20–24 years. For the socioeconomic eligibility criteria, most were confirmed to be living on a limited income (92.9%)—less than $20,000 CAD employment income annually, to have less than high school education (67.0%), and to be preparing to parent for the first time while single (90.9%). Regarding comparisons across the two age groups, women (20–24 years) reported statistically significantly higher educational attainment and income than girls (14–19 years) for both (*p* < 0.001). See Tables 4 and 5.

**Table 4:** Participant characteristics by eligibility criteria*

		Age Group		*p*-value
	Total *N* = 200	14–19 years *N* = 109	20–24 years *N* = 91	
	*n*/*N* (%)	*n*/*N* (%)	*n*/*N* (%)	
Living on less than $20,000 CAD	182/196 (92.9)	98/106 (92.5)	84/90 (93.3)	0.999
Having less than high school diploma	134/200 (67.0)	89/109 (81.7)	45/91 (49.5)	**< 0.001**
Preparing to parent while single	180/198 (90.9)	94/108 (87.0)	86/90 (95.6)	0.068

* Criteria used by public health nurses to screen and refer participants to study. N = number of respondents; n = number of respondents answering “yes”; CAD = Canadian dollars

**Table 5:** Sociodemographic characteristics*

		Age Group		*p*-value
	Total	14–19 years	20–24 years	
	*N* = 200	*N* = 109	*N* = 91	
	*n*/*N* (%)	*n*/*N* (%)	*n*/*N* (%)	
Cultural/ethnic background^†^				0.057
Indigenous including First Nations, Métis and Inuit	79/200 (39.5)	36/109 (33.0)	43/91 (47.3)	
Indigenous including First Nations, Métis and Inuit and other Ancestries	121/200 (60.5)	73/109 (67.0)	48/91 (52.7)	
Income from employment (annual CAD)				**< 0.001**
Less than $5,000	118/196 (60.2)	78/106 (73.6)	40/90 (44.4)	
$5,000–9,999	26/196 (13.3)	11/106 (10.4)	15/90 (16.7)	
$10,000–19,999	38/196 (19.4)	9/106 (8.5)	29/90 (32.2)	
$20,000–29,999	12/196 (6.1)	7/106 (6.6)	5/90 (5.6)	
$30,000 or more	2/196 (1.0)	1/106 (0.9)	1/90 (1.1)	
Highest educational qualification				**< 0.001**
Less than high school	134/200 (67.0)	89/109 (81.7)	45/91 (49.5)	
High school diploma or equivalent	53/200 (26.5)	18/109 (16.5)	35/91 (38.5)	
College or university	13/200 (6.5)	2/109 (1.8)	11/91 (12.1)	

* Data collected following study enrolment, during in-person baseline interviews. N = number of respondents; n = number of respondents answering “yes”; † Participants could give more than one answer. CAD = Canadian dollars

### Housing experiences

Most participants (89.6%) were living in a house, apartment or condominium, with only 10.4% living in less stable forms of housing such as group homes, shelters or single-room occupancy residences. The majority (63.3%) reported experiencing unstable housing (having moved three or more times or being homeless in the past year), with statistically significantly higher rates of instability reported by women (72.7%) than girls (55.6%; *p* = 0.02). See Table 6.

**Table 6:** Housing experiences*

		Age Group		*p*-value
	Total *N* = 200	14–19 years *N* = 109	20–24 years *N* = 91	
	*n*/*N* (%)	*n*/*N* (%)	*n*/*N* (%)	
Unstable housing				
Homeless ever (including currently)	109/194 (56.2)	52/105 (49.5)	57/89 (64.0)	0.059
Currently homeless	12/195 (6.2)	4/106 (3.8)	8/89 (9.0)	0.226
Moved ≥ 3 times or homeless (past year)	124/196 (63.3)	60/108 (55.6)	64/88 (72.7)	**0.020**
Current housing				0.692
House, apartment or condominium	173/193 (89.6)	95/106 (89.6)	78/87 (89.7)	
Group home, shelter or foster home	11/193 (5.7)	7/106 (6.6)	4/87 (4.6)	
Other (e.g., single-room occupancy residence)	9/193 (4.7)	4/106 (3.8)	5/87 (5.7)	

* Data collected following study enrolment, during in-person baseline interviews. N = number of respondents; n = number of respondents answering “yes”

### Health experiences

Regarding mental health, nearly one third of participants reported experiencing moderate-to-severe levels of psychological distress (29.3%), and nearly half reported severe anxiety or depression on a regular basis (48.5%). Less than one third (24.5%) self-reported being diagnosed with a mental disorder (e.g., attention problems) and even fewer (16.5%) reported diagnoses involving developmental conditions (e.g., learning disorders). Many reported being exposed to second-hand smoke in home and elsewhere (49.0%), with nearly one third reporting using cannabis at least once in the previous month (30.7%) or using nicotine/cigarettes in the previous two days (29.0%). Few reported using alcohol (7.5%) or street drugs (1.5%) in the previous month. Some participants also reported physical health concerns such as asthma or allergies (22.5%). There were no statistically significant differences across the two age groups. See Tables 7 and 8.

**Table 7:** Experiences of mental health including substance use*

		Age Group		*p*-value
	Total *N* = 200	14–19 years *N* = 109	20–24 years *N* = 91	
	*n*/*N* (%)	*n*/*N* (%)	*n*/*N* (%)	
Moderate-to-severe psychological distress	58/198 (29.3)	28/107 (26.2)	30/91 (33.0)	0.373
Mental health conditions^†^				
Severe anxiety or depression regularly	97/200 (48.5)	53/109 (48.6)	44/91 (48.4)	0.999
Diagnosed mental disorder (e.g., attention problems)	49/200 (24.5)	23/109 (21.1)	26/91 (28.6)	0.290
Diagnosed developmental conditions (e.g., learning disorders)	33/200 (16.5)	15/109 (13.8)	18/91 (19.8)	0.342
Mental health-substance use				
Nicotine/cigarette use (past two days)	58/200 (29.0)	32/109 (29.4)	26/91 (28.6)	0.999
Second-hand smoke exposure (past week)	98/200 (49.0)	55/109 (50.5)	43/91 (47.3)	0.757
Cannabis use (past month)	61/199 (30.7)	30/108 (27.8)	31/91 (34.1)	0.421
Alcohol use (past month)	15/199 (7.5)	6/109 (5.5)	9/90 (10)	0.355
Street drug use (past month)	3/200 (1.5)	2/109 (1.8)	1/91 (1.1)	0.999

* Data collected following study enrolment, during in-person baseline interviews. N = number of respondents; n = number of respondents answering “yes”; † Participants could give more than one answer

**Table 8:** Experiences of physical health*

		Age Group		*p*-value
	Total *N* = 200	14–19 years *N* = 109	20–24 years *N* = 91	
	*n*/*N* (%)	*n*/*N* (%)	*n*/*N* (%)	
Long-term physical health conditions^†^				
Asthma or allergies (regular use of puffers)	45 (22.5)	25 (22.9)	20 (22.0)	0.999
Iron-deficiency anemia	42 (21.0)	18 (16.5)	24 (26.4)	0.126
Other (e.g., serious injury with disability, thyroid disease)	58 (29.0)	33 (30.3)	25 (27.5)	0.999

* Data collected following study enrolment, during in-person baseline interviews. N = number of respondents; n = number of respondents answering “yes”; † Participants could give more than one answer

### Experiences of maltreatment

A high proportion—more than half the sample—were found to have a personal history of childhood maltreatment at age 16 years or younger (59.4%)—with statistically significantly higher rates reported by women (69.2%) than girls (50.6%; *p* = 0.014). Many also reported experiencing IPV in the past year (39.5%). See Table 9.

**Table 9:** Experiences of Maltreatment

		Age Group		*p*-value
	Total *N* = 200	14–19 years *N* = 109	20–24 years *N* = 91	
	*n*/*N* (%)	*n*/*N* (%)	*n*/*N* (%)	
Experiences of violence				
Maltreatment at age 16 years or younger	117/197 (59.4)	54/106 (50.9)	63/91 (69.2)	**0.014**
Intimate partner violence in past year	79/200 (39.5)	41/109 (37.6)	38/91 (41.8)	0.651

N = number of respondents; n = number of respondents answering “yes”

### Receiving health and social services

Of all participants, three quarters (75.0%) had received primary healthcare in the past month and more than one third (38.0%) had recently attended prenatal classes. One third (34.0%) had received income assistance in the past month through provincial or federal programs such as BC Income and Disability Assistance, Canada Disability Benefits and Employment Insurance, BC Hardship Assistance or BC Youth Agreements. Regarding comparisons across the two age groups, more women (48.4%) had received income assistance in the past month compared to girls (22%; *p* < 0.001). See Table 10.

**Table 10:** Receiving health and social services*

		Age Group		*p*-value
	Total *N* = 200	14–19 years *N* = 109	20–24 years *N* = 91	
	*n*/*N* (%)	*n*/*N* (%)	*n*/*N* (%)	
Health services for physical health				
Primary healthcare (past month)	150/200 (75.0)	83/109 (76.1)	67/91 (73.6)	0.806
Prenatal classes (past month)	76/200 (38.0)	42/109 (38.5)	34/91 (37.4)	0.981
Social services received				
Income assistance (past month)^†^	68/200 (34.0)	24/109 (22.0)	44/91 (48.4)	**< 0.001**

* Data collected following study enrolment, during in-person baseline interviews. N = number of respondents; n = number of respondents answering “yes”; † Participants could give more than one answer

### Cumulative adversities

We observed that almost all pregnant participants (99.5%) experienced two or more indicators of adversity, while most (80.5%) experienced four or more.

## Discussion

These data confirm that our recruitment processes reached and included a high proportion of urban, Indigenous girls and young women (27% in the trial versus 5.0% in the Canadian population [1])—despite no specialized study referral or recruitment pathways. We recognize the strengths of these mothers-to-be in seeking prenatal health services and in consenting to participate in a long-term research study—despite personal experiences of numerous adversities, barriers to accessing healthcare, and the legacies of harmful research and systemic racism that continue in present day [25, 36, 38, 59]. We acknowledge that the rates of adversities reported by these 200 mothers-to-be were higher than those rates reported by the full cohort of 729 girls and young women [44]. We present these descriptive data as a means of research inclusion—*ensuring that participants’ voices as told via the survey data*,* are included in research and knowledge dissemination* [20, 25]. Policymakers also have a responsibility for ensuring adequate services and can benefit from guidance on better reaching Indigenous girls and young women preparing to parent for the first time [60]. Our success in enrolling this population suggests that better service reach is indeed possible—and necessary—for Indigenous families in the Canadian context.

Our data also indicate that our relatively non-invasive socioeconomic screening criteria (i.e., being of young age, having limited access to education, living on low income, preparing to parent while single) were successful in reaching this cohort, who then reported experiencing associated cumulative adversities such as unstable housing and maltreatment experiences.

We found that young women demonstrated similar characteristics and experiences as did adolescent girls—beyond expected differences (e.g., more adolescents were still attending school). The majority of adolescent girls (81.7%) had not completed high school in early pregnancy and were likely to have their access to education interrupted. As others have suggested, young maternal age may not necessarily infer risk. Rather, maternal experiences of racial discrimination, combined with a lack of culturally-appropriate care, may be more predictive of child health and wellbeing, than young maternal age. [25, 36, 38, 61, 62] Indigenous Traditions may confer considerable supports to young mothers-to-be through extended family and community—covering wherever families reside [8, 9, 63, 64].

This study has several limitations. The trial, including recruitment, measures, data collection and the early prevention program being tested, was not designed by or for Indigenous Peoples, nor adapted to Indigenous methodologies and worldviews [65]. We did not collect data across distinct Indigenous groups, specifically First Nations, Métis and Inuit. As well, these data likely excluded those facing barriers to self-identification. Field interviewers involved in research data collection did not receive cultural-safety training. Further, our measures excluded some determinants of health and wellbeing particularly critical to Indigenous Peoples such as racism, as well as culture, identity, family and access to traditional lands. [5, 66, 67] With Indigenous co-leaders, we will include these topics in our planned long-term follow-up of this BCHCP trial cohort across middle childhood and adolescence—better ensuring that data describe the experiences of distinct First Nations, Métis and Inuit groups, and exploring facilitators and barriers to self-identification. Our data do not reflect the experiences of mothers-to-be residing in northern BC, as defined by BC regional HA boundaries. Northern HA has the largest and most diverse proportion of Indigenous Peoples and also the highest rates of maternal-child health disparities and inequitable access to health care compared to the rest of the province [68]. Another limitation to the current study, which is also a direction for future research, is the need for Indigenous-led research involving qualitative companion data exploring mothers’ experiences in more depth. Their stories, as told through research data, will help us better understand the structural, social and historical processes that give rise to health inequities in early pregnancy.

Our data suggest several directions for future research, beyond the BCHCP trial follow-up studies. Following wise practices of Indigenous Knowledges Translation, we will prepare a series of Indigenous-led, Indigenous-authored, and community-relevant published materials using the complete trial datase [69, 70]. We will facilitate exchange of Knowledges among nations, communities and Indigenous organizations—not ‘top down’ from researchers. We will apply the First Nations principles of data ownership, control, access and possession, ensuring the inclusion of young Indigenous mothers-to-be in the interpretation, presentation and co-authorship of community resources [71]. The new Knowledges generated by these lines of enquiry will focus on the strengths and traditional roles and responsibilities of the young Indigenous mothers and their children [72]. Facilitating this, our trial data repository contains longitudinal maternal-child data (observational and survey) collected from the 200 Indigenous mothers and their 237 children who were born to Indigenous mothers or fathers. The scientific team leading future work based on this data repository includes Indigenous co-leadership—overseeing all aspects of data governance, access and use. We will also engage with girls and young mothers throughout this work, for example through a project advisory group [71]. 

Overall, our findings underscore the need for new and enhanced investments in culturally-safe services that address avoidable adversities and their cumulative effects starting in early pregnancy [17, 29, 61, 62, 73]. We can better support communities in their own development and evaluation of maternal-child programs that they offer. These Indigenous participants were situated within a service delivery gap due to jurisdictional issues that create differential yet disadvantaged access to services on- and off-reserve. This research points to the need for targeted interventions that reach Indigenous girls and young women, particularly in urban environments. Such services can in turn promote healthy life trajectories involving child resiliency and wellbeing while also promoting maternal wellbeing—thereby further strengthening community [61, 64, 74, 75]. In particular, community-led prevention efforts that integrate Indigenous Knowledges with complementary Western research approaches such as the developmental origins of health and disease (DOHaD) have the potential to address childhood health inequities [76]. Indigenous Knowledges and DOHaD both acknowledge that children’s early experiences, including intergenerationally, are critical determinants of health and wellbeing across the lifecourse [76]. Crucially, both approaches also require an understanding of the historical origins of social inequities and the need for strength-based approaches involving longitudinal data collection with Indigenous children [75, 77–79]. In Canada and internationally, tailored Indigenous community-led programs are needed and are to be offered where Indigenous children and their mothers live [66, 74]—to support long-term mental health and well-being [5, 80].

## Conclusion

We conclude by expressing our respect and gratitude to the participants in our study. Our team of authors includes both Indigenous scholars and non-Indigenous researcher allies—all committed to addressing truth and reconciliation goals through research that can inform policy change. The participants and their willingness to engage in research despite the adversities they were dealing with have directly contributed to this process. Canada and BC must now urgently address the egregious avoidable adversities that stem from the persistent legacies and current experiences of colonialism—including racism, losses of lands and governance structures and rights, ongoing unacceptable socioeconomic disadvantage and the continuing intergenerational impact of residential schools including policies that targeted removal of children and the disruption of cultures, families, and communities. We celebrate the strength not only of the participants in this study, but also of Indigenous communities across Canada. Indigenous leadership in supporting mothers and children and including them in policy decisions will benefit all Canadians, while also forming a crucial step towards meaningful reconciliation. Alongside this, Indigenous-led research that includes the participants as partners and involves respectful collaborations—and is driven by community priorities—will also help in realizing the collective goal of ensuring healthy development for all Indigenous children.

## Data Availability

Data underlying the results reported here can be made available on a non-profit, cost-recovery basis upon reasonable request. We will share data with researchers who provide methodologically-sound proposals for review and who receive approval by the British Columbia (BC) Healthy Connections Project Data and Publication Committee, which comprises NC, HM, CW and JL, with consultation from other Indigenous scholars and BC’s First Nations Health Authority and Mètis Nation BC, where applicable. This Committee is governed by the Project’s Data and Publication Agreement, which applicants will be required to sign prior to data release. We will share data via secure electronic file transfer. Priority will be given to researchers with interests and expertise whose aims overlap with the Project, which has focused on maternal-child health and wellbeing. For data sharing enquiries, contact the corresponding author (NC).

## Abbreviations

BC: British Columbia
FNHA: First Nations Health Authority
CAD: Canadian Dollars
BCHCP: British Columbia Healthy Connections Project
LHA: Local Health Area
HA: Health Authority
MNBC: Mètis Nation of British Columbia
SFU: Simon Fraser University
IPV: Intimate Partner Violence
DOHaD: Developmental Origins of Health and Disease
